# Supplementary figures and images for: Interactive effects between soil properties and bacterial communities in tomato rhizosphere under the application of microbial agents
Source: PeerJ. 2025 Jun 30;13:e19564. doi: 10.7717/peerj.19564 (PMC12225625; doi:10.7717/peerj.19564)

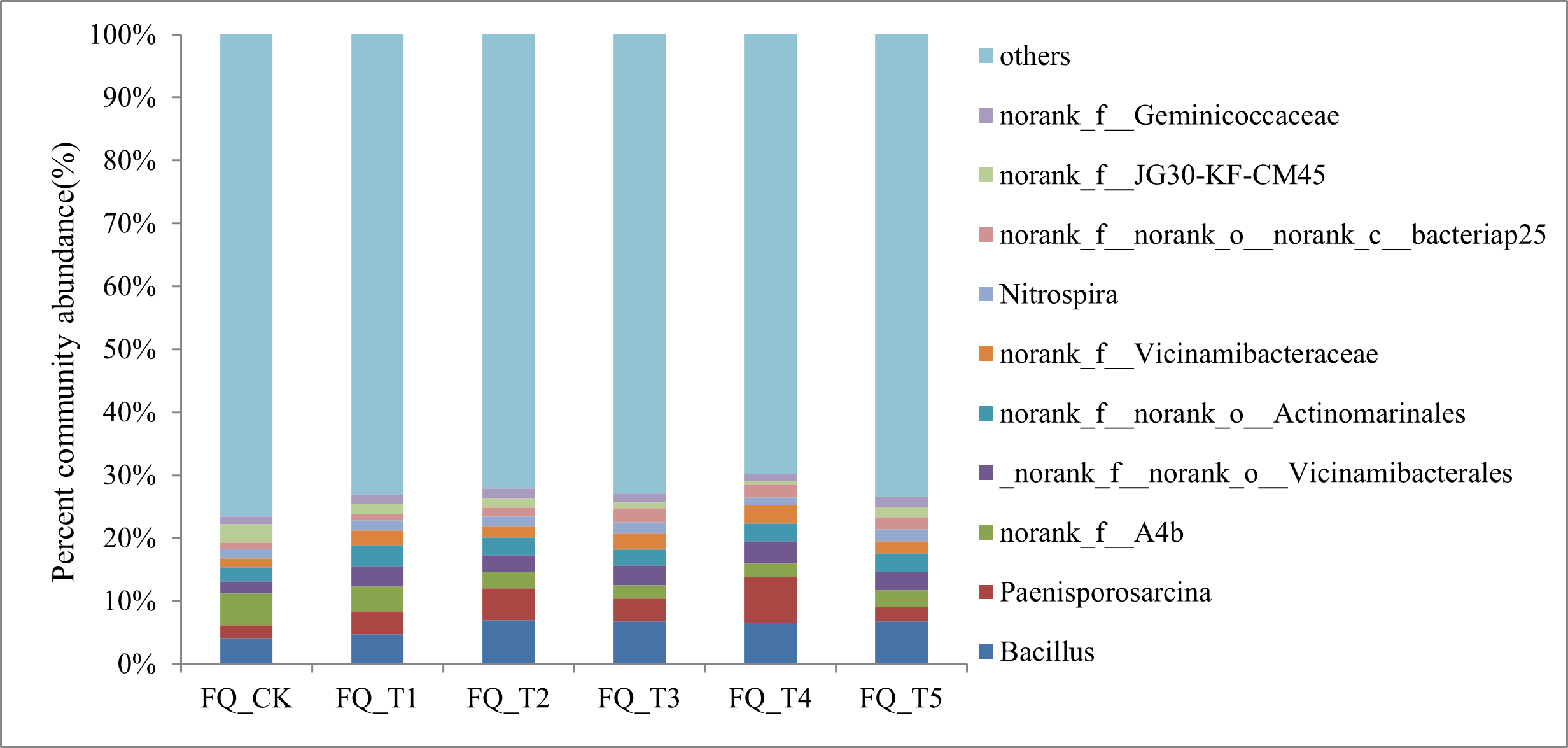

Supplement: Supplemental Information 7 [file peerj-13-19564-s007.png]
